# Photobiomodulation and low-intensity pulsed ultrasound synergistically enhance dental mesenchymal stem cells viability, migration and differentiation: an *invitro* study

**DOI:** 10.1007/s10266-024-00920-6

**Published:** 2024-03-22

**Authors:** Mohamed Shamel, Shereen Raafat, Ikhlas El Karim, Shehabeldin Saber

**Affiliations:** 1 Department of Oral Biology, Faculty of Dentistry, The British University in Egypt, El Sherouk City, Egypt; 2 Department of Pharmacology, Faculty of Dentistry, The British University in Egypt, El Sherouk City, Egypt; 3 Dental Science Research Group, Health Research Centre of Excellence, The British University in Egypt (BUE), El Sherouk City, Egypt; 4 School of Medicine, Dentistry and Biomedical Sciences, Queen’s University, Belfast, UK; 5 Department of Endodontics, Faculty of Dentistry, The British University in Egypt, El Sherouk City, Egypt

**Keywords:** Mesenchymal stem cells, Photobiomodulation, Low-intensity pulsed ultrasound, Osteogenesis, Odontogenesis

## Abstract

Novel methods and technologies that improve mesenchymal stem cells (MSCs) proliferation and differentiation properties are required to increase their clinical efficacy. Photobiomodulation (PBM) and low-intensity pulsed ultrasound (LIPUS) are two strategies that can be used to enhance the regenerative properties of dental MSCs. This study evaluated the cytocompatibility and osteo/odontogenic differentiation of dental pulp, periodontal ligament, and gingival MSCs after stimulation by either PBM or LIPUS and their combined effect. MTT assay, cell migration assay, osteo/odontogenic differentiation by AR staining and ALP activity, and expression of osteo/odontogenic markers (OPG, OC, RUNX2, DSPP, DMP1) by RT-qPCR were evaluated. Statistical analysis was performed using ANOVA, followed by Tukey’s post hoc test, with a *p*-value of less than 0.05 considered significant. The results showed that combined stimulation by PBM and LIPUS resulted in significantly the highest viability of MSCs, the fastest migration, the most dense AR staining, the most increased ALP activity, and the most elevated levels of osteogenic and odontogenic markers. The synergetic stimulation of PBM and LIPUS can be utilized in cell-based regenerative approaches to promote the properties of dental MSCs.

## Introduction

Tissue engineering is a rapidly growing popular discipline of bioscience that aims to regenerate damaged or lost tissues [1]. Ongoing research and technological developments in biomaterial science and tissue engineering have significantly advanced regenerative dentistry. Unlike conventional dental treatment, this interdisciplinary approach aims to revive and maintain the biological vitality of tissues that have been lost or injured [2]. Three components are essential for successful cell-based tissue engineering: cells, scaffolds, and growth factors or signaling molecules [3]. Hence, carefully choosing methods for cell stimulation, scaffold production, and tissue transplantation is crucial in determining the outcome of cell-based tissue engineering endeavors. Furthermore, the practical efficacy of the components depends on their suitable combination and the functional synergy among them [4, 5].

Mesenchymal stem cells (MSCs) are the most commonly used cells in tissue regeneration of the craniofacial complex. They possess self-renewal capacity, multipotency, immunomodulatory, angiogenic, and antioxidative properties [6, 7]. Bone marrow MSCs (BMMSCs) have traditionally been regarded as the preferred type of MSCs for tissue engineering purposes. Nevertheless, invasive harvesting techniques and the restricted number of cells obtained are notable disadvantages.

On the other hand, MSCs derived from oral sources are considered easily accessible and have abundant cell yields. Various subpopulations of dental MSCs (DMSCs) have been studied, including dental pulp (DPSCs), periodontal ligament (PDLSCs), and gingival (GMSCs). DMSCs have been used in many applications in craniofacial tissue engineering, such as dental tissue regeneration [8], regenerative endodontic therapies [9], bone and cartilage regeneration [10], nerve regeneration [11], wound healing [12], and immune-mediated disorders [13]. Nevertheless, the accessibility of these resources is contingent upon the isolation of extracted wisdom teeth, gingival biopsies, or undesired teeth resulting from orthodontic procedures. This approach necessitates, to some degree, intricate procedures along with the potential contamination hazard. Furthermore, the productivity, growth, and ability of DMSCs to differentiate into tooth-related structures decrease with age [14, 15]. Moreover, the yield of DPSCs and PDLSCs is restricted compared to other MSCs, thereby impacting the effectiveness of dental MSC-based treatments. New techniques and technologies are needed to enhance these cells' ability to multiply and specialize to improve their effectiveness in medical applications.

One of these techniques is photobiomodulation (PBM) or low-level laser therapy (LLLT). PBM uses low-powered, visible, and near-infrared light from different light sources to affect biological processes. When absorbed by endogenous chromophores, light within specific wavelength ranges is a form of non-ionizing electromagnetic radiation that can trigger photophysical and photochemical responses. Pain alleviation, triggering in vitro and in vivo tissue regeneration, and stimulating cell proliferation and healing are only some of the nondestructive photobiological activities that can be activated by PBM's use of light at relatively low intensity [16, 17]. The positive effect of PBM on MSCs has been well-documented in many studies. Yoo et al. [18] revealed that PBM promotes the differentiation, proliferation, and migration of MSCs. Moreover, many previous studies have shown the enhancement of osteogenic [19, 20] and odontogenic [21, 22] differentiation of MSCs following PBM therapy.

Ultrasound (US) energy is a state-of-the-art approach to improve MSC-based therapy. Low-intensity pulsed ultrasound (LIPUS) is a unique method that releases bursts of low-intensity waves. This procedure is nonintrusive, safe, and simple, and it serves as a physical treatment technique that can cause mechanical, cavitation, and thermal effects [23]. Scientific data supports the efficacy of LIPUS in promoting the growth, specialization, and mobility of MSCs [24, 25]. In addition, LIPUS has effectively addressed medical issues, including fractures, arthritis, and damage to tendons and ligaments.

The effect of PBM and LIPUS on MSCs of oral origins when used in isolation is documented, but their combined effect has yet to be discovered. Emerging evidence suggests that combined laser and US techniques demonstrated significant advantages over pure US-based and laser-based thrombolysis [26]. Furthermore, new laser-ultrasonic combinations have been developed for cancer treatment [27]. Therefore, we assume that the combined use of PBM and LIPUS enhanced dental MSCs viability, migration, and differentiation capacity.

Therefore, this research examined how PBM and LIPUS affected DPSCs, PDLSCs, and GMSCs. This study's findings can be used to develop a preconditioning regimen for cells prior to transplantation for dental pulp tissue engineering. The null hypothesis being tested is that there is no difference in the viability, migration, and differentiation of DPSCs, PDLSCs, and GMSCs after stimulation by PBM, LIPUS, or their combination.

## Materials and methods

### Cell isolation and culture

Approval for the experimental design and procedures (FD BUE REC 22-027) was provided by the Research Ethics Committee of the Faculty of Dentistry at The British University in Egypt. All subjects and/or their legal guardian(s) granted informed consent to use their extracted teeth for scientific purposes. We extracted and gathered impacted third molars (*N* = 5) from healthy donors between the ages of 20 and 40. All experimental procedures were performed in the cell culture laboratory at the British University in Egypt. Pulp, periodontal ligament, and gingival tissues were acquired from the extracted molars. These tissues were subsequently sliced into small pieces, and stem cells were extracted using the enzymatic digestion method, as previously described [28]. In brief, tissue fragments from the three sources were placed individually in phosphate-buffered saline (PBS) (Gibco BRL, CA, USA) containing 3 mg/ml collagenase I and 4 mg/ml dispase II (Sigma, St. Louis, MO, USA) and incubated for 45 min in a shaking water bath at 37 °C. Following PBS filtration, cell pellets were obtained through centrifugation and cultured in 1 ml of complete culture medium, which consisted of DMEM/F12 (Dulbecco’s modified Eagle’s medium/F-12 Ham medium, Sigma) conditioned with 10% fetal bovine serum (FBS, Gibco, Grand Island, NY, USA). Upon reaching 90% confluence, cell subculturing was performed. The morphology of the isolated cells was observed under an inverted microscope (Olympus, Tokyo, Japan). Cells from the fourth passage were utilized for this study [29].

### Stem cells characterization

To characterize the dental MSCs cultures, the detection of surface antigens and examination of the multilineage differentiation potential were performed. Flow cytometry analysis was performed to detect the surface antigens CD34, CD45, CD90, CD105, CD73, and HLA-DR using a flow cytometer (Cytofex, Beckman Coulter) [30]. Next, we assessed the multilineage differentiation potential of the isolated stem cells. To briefly describe the process, cells were cultured in 12-well plates at a density of 10 × 10^4^ cells per well in a culture medium supplemented with the Adipo-Chondro-Osteo differentiation kit (Human mesenchymal stem cell functional identification kit, R and D Systems, Minneapolis, USA). The culture medium was refreshed twice a week, and after the differentiation period, we performed staining in the wells to identify specific products resulting from the differentiated cells. Oil Red stain, Alizarin Red S stain, and Alcian Blue stain (Sigma Aldrich, Steinheim, Germany) were used to examine adipogenic, osteogenic, and chondrogenic differentiation, respectively [29].

### Grouping

For each cultured cell type, DPSCs, PDLSCs, and GMSCs, groups were assigned as follows:

#### Control

MSCs without any intervention, serving as a negative control.

#### Osteogenic group

MSCs cultured in osteogenic media, serving as a positive control.

#### PBM

MSCs subjected to PBM irradiation in a single dose for 60 s using a GaAIAs Diode laser device (K2 mobile laser, Hulaser, Seoul, Republic of Korea), wavelength 980 nm, power output 100 mW, in continuous mode, and energy density of 3 J/cm^2^. The distance between the well surface and the laser spot was 0.5 cm [31].

#### LIPUS

MSCs subjected to LIPUS application using an ultrasound device (Endo One, Guilin Woodpecker, Guangxi, China) with the following parameters (frequency 45 ± 4 kHz, intensity, intensity 750 mW/cm2, and pulse duration 1/45000 s). The tip of the LIPUS device was carefully positioned to be in contact with the surface of the culture medium without touching the bottom of the well for 5 min [32].

#### PBM + LIPUS

MSCs subjected to PBM followed by LIPUS.

### MTT assay

Cell viability was assessed on days 2 and 6 through the MTT assay (*n* = 3). In each well of a 6-well plate, 30 × 10^4^ cells from the three cell sources were seeded with culture medium and subsequently incubated at 37 °C with 5% CO_2_. The following day, the cells were subjected to one of three conditions: laser exposure, LIPUS exposure, or a combination of both (cells exposed to both laser and LIPUS). On days 2 and 6, the culture medium was aspirated, and 1 ml of 3-(4, 5-dimethylthiazol-2-yl)-2, 5-diphenyltetrazolium bromide (MTT) reagent (Sigma Aldrich, Steinheim, Germany) in PBS was added, followed by a 4-h incubation period. The resultant violet formazan crystals were dissolved using dimethyl sulfoxide (DMSO, Sigma, St. Louis, MO, USA), and the light absorption value was measured using a microplate reader at 570 nm. The values obtained from each well were standardized against the control group (cells with medium only), and the results were expressed as a percentage of viability using the following equation [33]: Cell viability % = Absorbance of test/Absorbance of control × 100.

### Cell migration assay

Cell migration ability was evaluated using the scratch wound assay (*n* = 3). As in the MTT assay, cells were seeded in a 6-well plate in a complete culture medium, incubated at 37 °C, and 5% CO_2_ until cells reached 100% confluence. All media were removed, and a scratch was induced in each well using a sterile 200 μm pipette tip. The wells were then washed carefully with PBS to remove cell debris, a culture medium was added, and the cells were exposed to the laser, LIPUS, and a combination of both. Each well's initial scratch (day 0) was imaged using a phase contrast microscope (TCM 400, LABOMED, USA), and images were obtained at the same scratch area after 1, 2 and 3 days. The wound area and closure percentage were calculated for each well using ImageJ software (National Institutes of Health, Bethesda, MD, USA) [34].

### Osteogenic differentiation

Cells were initially seeded into a 6-well plate at a density of 30 × 10^4^ cells per well (*n* = 3). The culture medium was replaced with the osteogenic medium to induce osteogenic differentiation. This specialized medium consisted of a complete culture medium supplemented with 0.1 μM dexamethasone, 2.5 mg/l l-ascorbic acid (both obtained from Sigma Aldrich, Steinheim, Germany), and 10 mM beta-glycerophosphate (obtained from Merck, Darmstadt, Germany). Subsequently, the cells were subjected to a single exposure of either laser, LIPUS, or a combined stimulation of both methods. Throughout two weeks, the osteogenic medium and the regular culture medium were changed every three days.

It is important to note that cells grown in complete culture medium served as the negative control group, whereas cells cultured in osteogenic medium were considered the positive control group.

### Alizarin Red S assay

The Alizarin Red assay was conducted after the emergence of mineralized nodules following two weeks of osteogenic differentiation (*n* = 3). In a concise description of the procedure, cells were rinsed and fixed utilizing 70% (v/v) ethanol for 15 min. A solution containing 40 mM Alizarin Red S (obtained from Sigma-Aldrich, Steinheim, Germany) was introduced and incubated for 30 min at room temperature. After removing the excess stain, an inverted microscope was employed to capture images of the mineralized nodules. To transform the red dye into a yellow hue, 10% glacial acetic acid (obtained from Sigma-Aldrich, Steinheim, Germany) was used for solubilization. The converted solution was measured for absorbance at 405 nm using a microplate reader. The negative control group consisted of cells cultivated in a standard medium, whereas the positive control group comprised cells produced in an osteogenic media [35].

### Alkaline phosphatase assay

The activity of the alkaline phosphatase (ALP) enzyme in the cultivated cells was evaluated using an ALP assay (*n* = 3). Following the osteogenic differentiation process, the wells were subjected to two rounds of washing using alkaline phosphatase buffer (ALPB). Subsequently, each well received an addition of para-nitrophenyl phosphate (p-NPP) at a concentration of 1 mg/ml, and this was mixed with an equal volume of ALPB (both acquired from Sigma-Aldrich, Steinheim, Germany). 50 μL of the mixture was aspirated and combined with 1 M sodium hydroxide (NaOH) to halt the reaction. At the initiation of the reaction (time zero), the absorbance of the aspirated solution was measured at 405 nm using a microplate reader. This measurement procedure was repeated for each well every minute over a span of 10 min to capture the progressive development of a yellow color resulting from the accumulation of p-nitrophenolate (p-NP), a byproduct of ALP activity. Subsequently, the absorbance values were plotted against time, enabling the calculation of the reaction slope for each well. This slope was indicative of the reaction rate of each [35].

### Osteogenic and odontogenic markers detection using RT-qPCR

Quantification of osteogenic and odontogenic gene expression was accomplished through RT-qPCR analysis (*n* = 3) of key genes, including *Runt-related transcription factor 2* (*RUNX2*), *osteoprotegerin* (*OPG*), *osteocalcin* (*OC*), *dentin matrix protein-1* (*DMP1*), and *dentin sialophosphoprotein* (*DSPP*). In a brief overview of the procedure, total mRNA extraction from each sample was carried out using the QIAGEN RNA Extraction kit (QIAGEN GmbH, Hilden, Germany) according to the manufacturer's guidelines and subsequent cDNA synthesis. The resulting cDNA was amplified and quantified using SYBR Green Supermix (Bio-Rad) on an RT-qPCR machine from Bio-Rad. The measured mRNA expression levels were normalized in relation to β-actin. The sequences of the primers used for the osteogenic and odontogenic markers can be found in Table 1 [29]. All samples were measured in triplicate and expressed as fold change relative to the control.

**Table 1:** Osteogenic and odontogenic marker primer sequences

Gene	Forward sequence	Reverse sequence
*OPG*	*5ˊ-CTAATTCAGAAAGGAAATGC -3ˊ*	*5ˊ- GCTGAGTGTTCTGGTGGACA -3ˊ*
*OC*	*5ˊ- TTCATGTGGGGTGTCTCTGA -3ˊ*	*5ˊ- CTGGGCCTTGGTCTTGAGT- 3ˊ*
*RUNX2*	*5ˊ- GTTATGAAAAACCAAGTAGCCAGGT -3ˊ*	*5ˊ- GTAATCTGACTCTGTCCTTGTGGAT- 3ˊ*
*DMP1*	*5′-AGGAAGTCTCGCATCT CAGAG-3’*	*5′-TGGAGTTGCTGTTTTCTGTAGAG-3’*
*DSPP*	*5′-TCACAAGGGAGAAGGGAATG-3′*	*5′-TGCCATTTGCTGTGATGTTT-3′*
*β-actin*	*5ˊ TCCGTCGCCGGTCCACACCC-3ˊ*	*5ˊ-TCACCAACTGGGACGATATG- 3ˊ*

### Statistical analysis

The outcomes of the MTT assay, cell migration assay, and osteo/odontogenic differentiation data are presented as the mean values accompanied by their respective standard deviations. Significance in statistical terms was evaluated between the treatment and control groups and between the osteogenic groups (positive control) for each cell type. Additionally, statistical comparisons were made among the different cell stimulation methods: PBM, LIPUS, and combined stimulation. All sets of samples underwent a normality test using the Shapiro–Wilk test. The subsequent data analysis involved ANOVA followed by Tukey’s post hoc test, with statistical significance defined as *p* < 0.05. The software employed for these statistical analyses was GraphPad Prism 9.0 (GraphPad Software, San Diego, CA, USA). All experiments were repeated in triplicate. The studies were replicated three times, and each sample was measured in triplicate (*n* = 3).

## Results

### Stem cell characterization

#### Isolation and culture

**Fig. 1:**
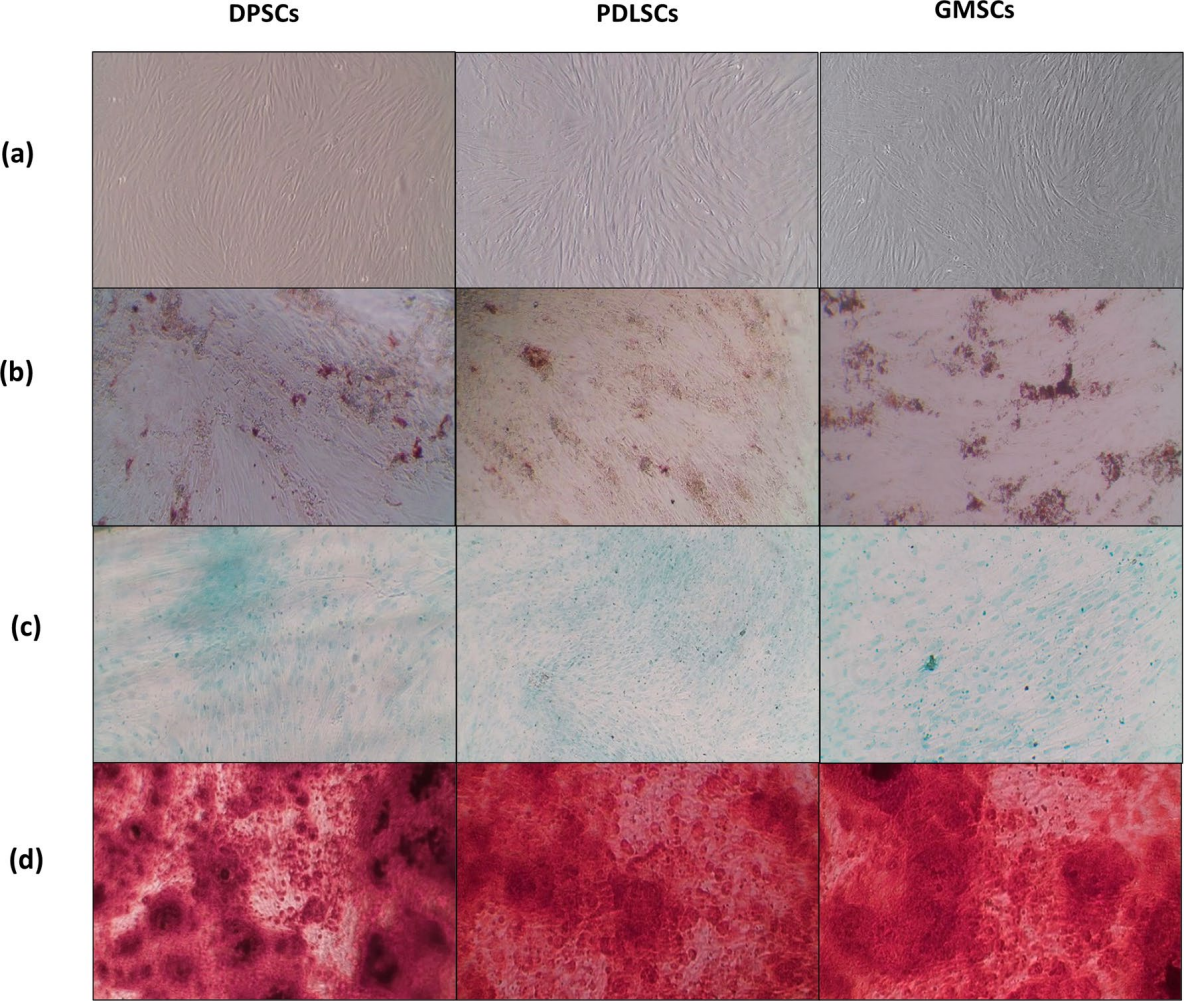
**a** Photomicrographs showing DPSCs, PDLSCs, and GMSCs cell morphology at passage 4. **b** Adipogenic differentiation confirmed by Oil Red O staining. **c** Chondrogenic differentiation was confirmed by Alcian blue staining. **d** Osteogenic differentiation confirmed by Alizarin red staining

DPSCs, PDLSCs, and GMSCs were successfully isolated by enzymatic digestion and amplified by adherence separation, reaching 80% confluence by day 14. Cells observed using an inverted light microscope displayed characteristics of stem cells, including an adherent and elongated phenotype, as well as proliferation in the form of colonies (Fig. 1a).

#### Multilineage differentiation

After culture in adipogenic, chondrogenic, and osteogenic media, all MSCs showed tri-lineage differentiation potential, as confirmed by morphological changes and special stains (Fig. 1b–d).

### Flow cytometry

**Fig. 2:**
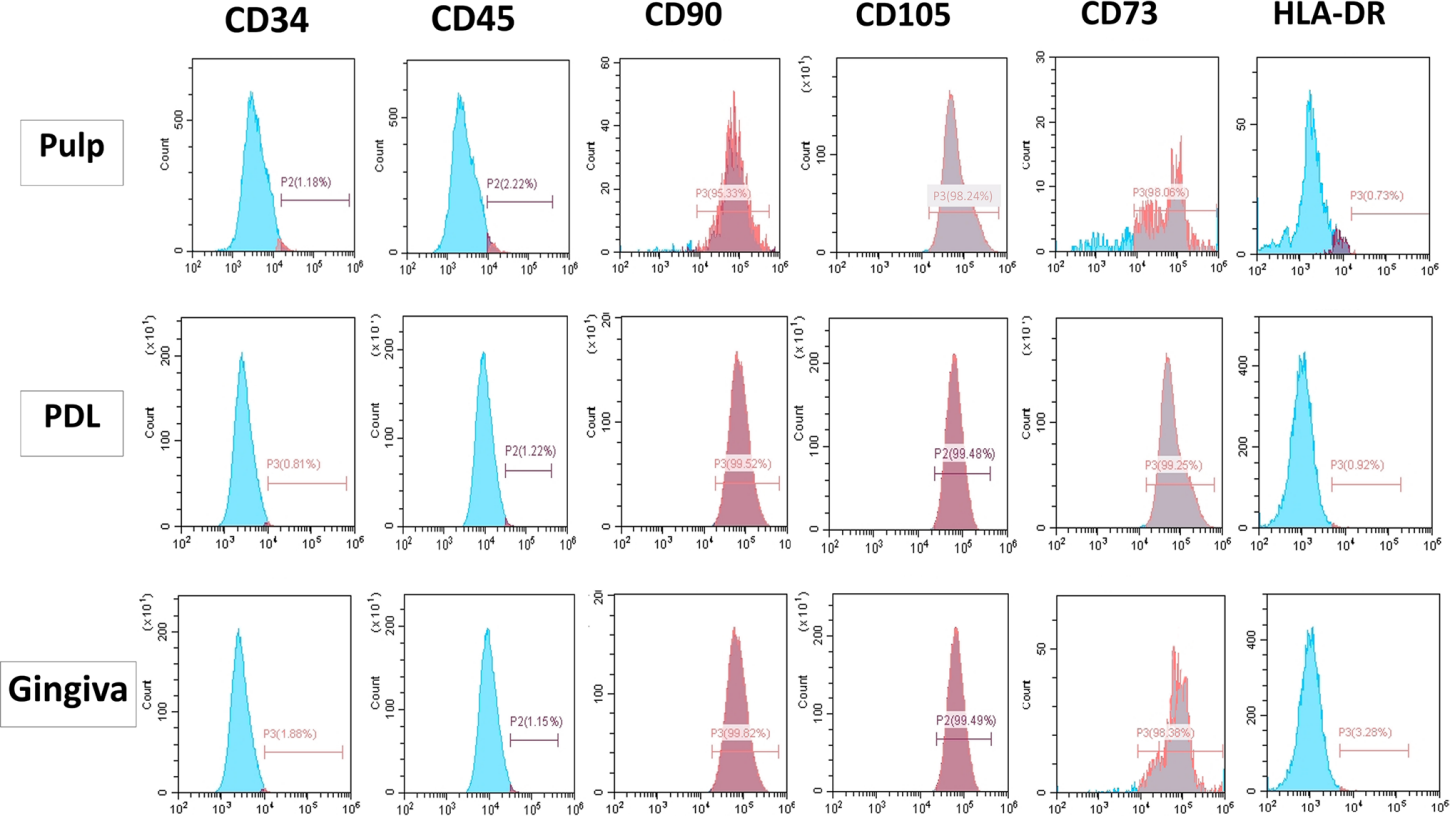
Expression of cell surface markers of DPSCs, PDLSCs, and GMSCs by flow cytometric analysis

DPSCs, PDLSCs, and GMSCs showed positive expression of the MSCs-specific markers CD 73, CD90, and CD105. At the same time, they were negative for the hematopoietic stem cell markers CD34, CD45, and HLA-DR, indicating that these cells have MSCs properties (Fig. 2).

### MTT assay

MTT results on day 2 indicate that LIPUS treatment increased the MTT assay values for DPSCs. PDLSCs and GMSCs showed little change compared to the control group. PBM treatment also increased MTT assay values for DPSCs and GMSCs, with a more modest effect on PDLSCs. The combined LIPUS and PBM treatment had a notable impact on GMSCs, while its effects on DPSCs and PDLSCs were less pronounced.

**Fig. 3:**
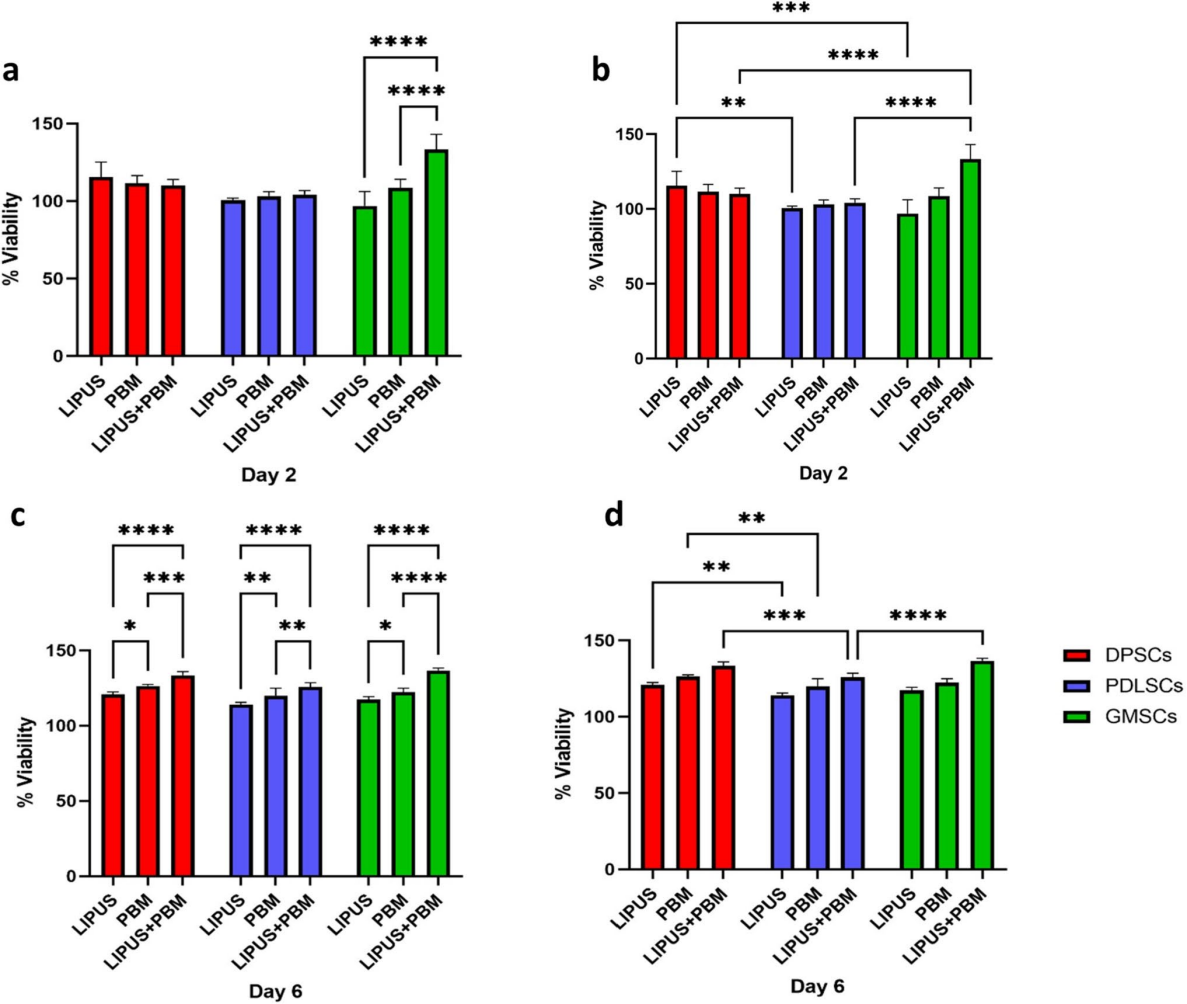
Mean and standard deviation of cell viability percentage after MTT assay, and comparative statistical results on day 2 (**a**) intragroup comparison, (**b**) intergroup comparison, and day 6 (**c**) intragroup comparison, (**d**) intergroup comparison. *p < 0.05, **p < 0.01, ***p < 0.001, ****p < 0.0001 indicate significance for each group compared to the control group and differences between the same stimulation method(s) of groups

On day 6, LIPUS treatment continued to increase the MTT assay values for all three cell types, with GMSCs showing the highest values. PBM treatment enhanced the MTT assay values for all cell types, with DPSCs having the highest values. The combined LIPUS and PBM treatment maintained or further increased the MTT assay values for all three cell types, with GMSCs exhibiting the highest values again. The mean and standard deviation of the MTT assay and the statistical comparisons are shown in Fig. 3.

The pattern of MTT assay results in terms of treatment effectiveness remains consistent between day 2 and day 6 for each stem cell type. On both days, LIPUS + PBM consistently appeared to be the most effective treatment in enhancing cell viability, followed by PBM and then LIPUS alone in most cases. The differences between treatment groups tended to become more significant on day 6 compared to day 2, suggesting that the effects of the treatments on cell viability may become more pronounced over time.

### Wound healing assay (cell migration)

**Fig. 4:**
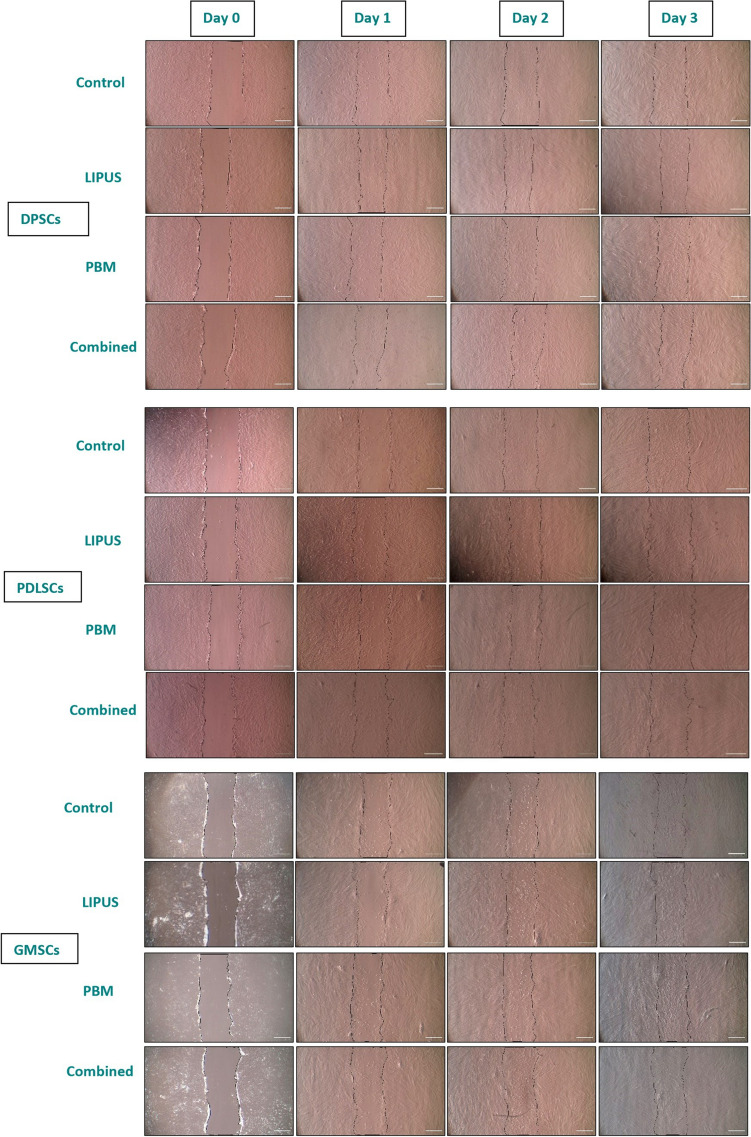
Photomicrographs showing cell migration assays at days 0 (scratch initiation), 1, 2 and 3

A cell migration assay was performed, and the wound closure percentage was measured on days 1, 2, and 3 (Fig. 4). On day 1, among DPSCs, PBM and LIPUS + PBM showed slightly higher wound closure percentages than the control, but the differences were not statistically significant (*p* > 0.05). Within PDLSCs, all treatment groups (LIPUS, PBM, LIPUS + PBM) showed significantly higher wound closure percentages compared to control (*p* < 0.05). Within GMSCs, there were no significant differences (*p* > 0.05) between the treatment groups. On day 2, the combined stimulation method showed the highest wound closure percent, which was statistically significant than the control group in the DPSCs (*p* < 0.0001), PDLSCs (*p* = 0.0024), and GMSCs (*p* < 0.0001) groups. In addition, on day 2, GMSCs stimulated with the combined methods showed the highest wound closure percentage compared to the other groups. Moreover, the combined method showed a higher significant difference when compared to PBM and LIPUS stimulation alone in both the DPSCs (*p* = 0.0024, *p* < 0.0001) and GMSCs (*p* = 0.0167, *p* < 0.0001) groups and a nonsignificant difference in the PDLSCs group (*p* = 0.685, *p* = 0.986).

In addition, on day 3, combined stimulation of DPSCs showed a significantly (*p* = 0.0156) higher wound closure compared to control and LIPUS (*p* = 0.0013), while a non-significant (*p* = 0.793) difference was found when compared to PBM. Similar results were found in PDLSCs, where the combined stimulation showed a significant difference when compared to control (*p* = 0.011) and LIPUS (*p* = 0.018) and a non-significant (*p* = 0.729) difference when compared to PBM. Combined stimulation of GMSCs showed a significant difference (*p* = 0.04) compared to the control and a nonsignificant difference compared to LIPUS (*p* = 0.091) and PBM (*p* = 0.991). A nonsignificant difference (*p* > 0.05) was found between the control and LIPUS and PBM groups in all cell types.

The mean and standard deviation of the wound closure percentage and comparative statistical analysis are shown in Table 2.

**Table 2:** Mean and standard deviation of wound closure percentage (%) at days 1, 2, and 3 after the cell migration assay

	DPSCs	PDLSCs	GMSCs
	Mean% ± Std	Mean% ± Std	Mean% ± Std
Day 1			
Control	49.0 ± 6.5	55.0^A^ ± 5.3	50.6 ± 4.2
LIPUS	49.0^a^ ± 3.3	61.7^Bb^ ± 3.0	51.3^a^ ± 2.6
PBM	52.3^a^ ± 5.0	63.0^Bb^ ± 4.7	58.2^ab^ ± 3.5
LIPUS + PBM	55.1^a^ ± 3.2	67.8^Bb^ ± 3.8	59.5^ab^ ± 2.8
Day 2			
Control	80.8^Aa^ ± 1.7	80.3^Aa^ ± 3.5	71.3^Ab^ ± 2.8
LIPUS	82.0^A^ ± 3.1	85.5^AB^ ± 3.9	83.2^BC^ ± 2.3
PBM	84.5^A^ ± 2.7	84.0^AB^ ± 1.7	85.9^C^ ± 1.9
LIPUS + PBM	91.6^B^ ± 1.1	87.4^B^ ± 1.7	92.0^D^ ± 1.5
Day 3			
Control	96.5^A^ ± 0.4	96.3^A^ ± 0.1	96.6^A^ ± 0.0
LIPUS	96.0^A^ ± 2.1	96.5^A^ ± 1.9	97.1^AB^ ± 1.3
PBM	98.4^AB^ ± 1.3	98.2^AB^ ± 1.6	98.9^AB^ ± 1.5
LIPUS + PBM	100^B^ ± 0	100^B^ ± 0	100^B^ ± 0

Different superscript capital letters in the same column indicate significant differences (*p* < 0.05); different superscript small letters in the same row indicate significant differences (*p* < 0.05)

### Alizarin Red

**Fig. 5:**
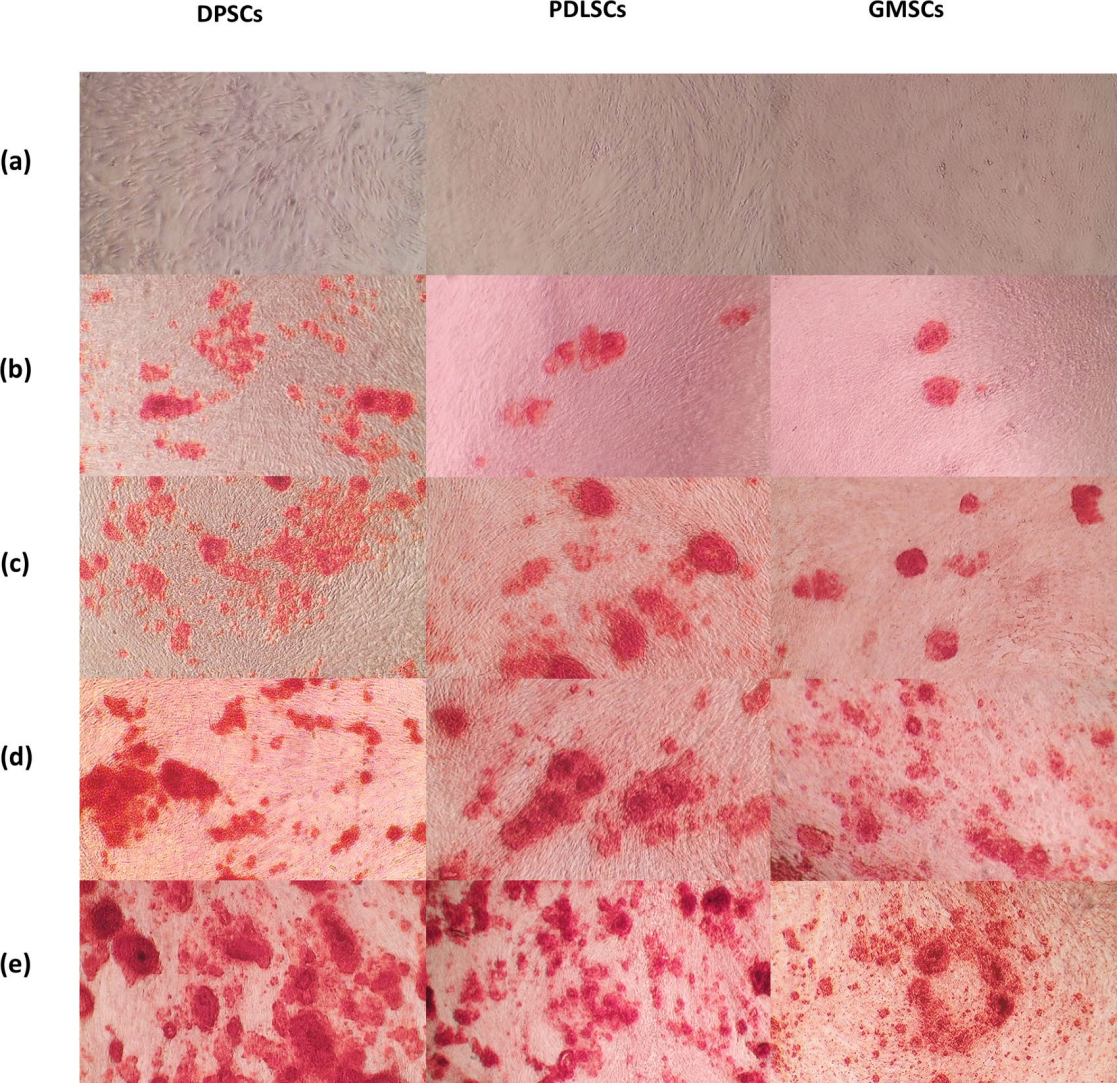
Alizarin red staining of DPSCs, PDLSCs, and GMSCs after 2 weeks of culturing in osteogenic media; (**a**) control, (**b**) osteogenic media, (**c**) LIPUS, (**d**) PBM and (**e**) LIPUS and PBM combination group

Alizarin red staining analysis of cells from each experimental group on day 14 showed a positive reaction, indicating the formation of calcified nodules. The combined activation method within each stem cell type had the highest staining among all groups (Fig. 5). Quantitative analysis of Alizarin Red staining showed that, in the DPSCs group, the combined approach showed significantly higher staining than LIPUS (*p* < 0.0001) and PBM (*p* < 0.0001). Moreover, laser irradiation showed a significantly (*p* < 0.0001) higher difference in DPSCs than LIPUS. In the PDLSCs group, the combined method was significantly (*p* < 0.05) higher than all other groups. A nonsignificant (*p* = 0.078) difference was found between the LIPUS and PBM groups of PDLSCs. In the GMSCs group, the combined method was significantly (*p* < 0.05) higher than all other groups of the same cell line, while a significant (*p* < 0.0001) difference was found between the LIPUS and PBM groups. Comparing stimulating methods among different cell types showed that LIPUS stimulation and laser irradiation of DPSCs were significantly (*p* < 0.05) higher than PDLSCs and GMSCs. Furthermore, the combined approach of stimulation of DPSCs had a statistically higher significance than PDLSCs (*p* < 0.0001) and GMSCs (*p* < 0.0001) groups. Moreover, combined stimulation with GMSCs had a statistically higher significance (*p* < 0.0001) than PDLSCs. The average absorbance rates for Alizarin red staining and statistical analysis of the average absorbance rate of all groups are summarized in Table 3.

**Table 3:** Mean and standard deviation of Alizarin Red S staining

	DPSCs	PDLSCs	GMSCs
	Mean absorbance at 405 nm ± StD	Mean absorbance at 405 nm ± StD	Mean absorbance at 405 nm ± StD
Control	0.066^A^ ± 0.0009	0.065^A^ ± 0.006	0.059^A^ ± 0.001
Osteogenic media	0.222^B^ ± 0.01	0.214^B^ ± 0.007	0.201^A^ ± 0.003
LIPUS	0.469^Ca^ ± 0.012	0.351^Cb^ ± 0.006	0.345^Bb^ ± 0.015
PBM	0.770^Da^ ± 0.027	0.384^Cb^ ± 0.003	0.526^Cc^ ± 0.045
LIPUS + PBM	0.957^Ea^ ± 0.441	0.561^Db^ ± 0.055	0.830^Dc^ ± 0.101

Different superscript capital letters in the same column indicate significant differences (*p* < 0.05); different superscript small letters in the same row indicate significant differences (*p* < 0.05)

### ALP activity

The results demonstrated that DPSCs are generally the most responsive to the various treatments, followed by PDLSCs, with GMSCs exhibiting the lowest ALP activity. The combined therapy of LIPUS and PBM appears to have the most pronounced effect on enhancing ALP activity, indicating its potential for accelerating osteogenic differentiation in these stem cell types.

**Fig. 6:**
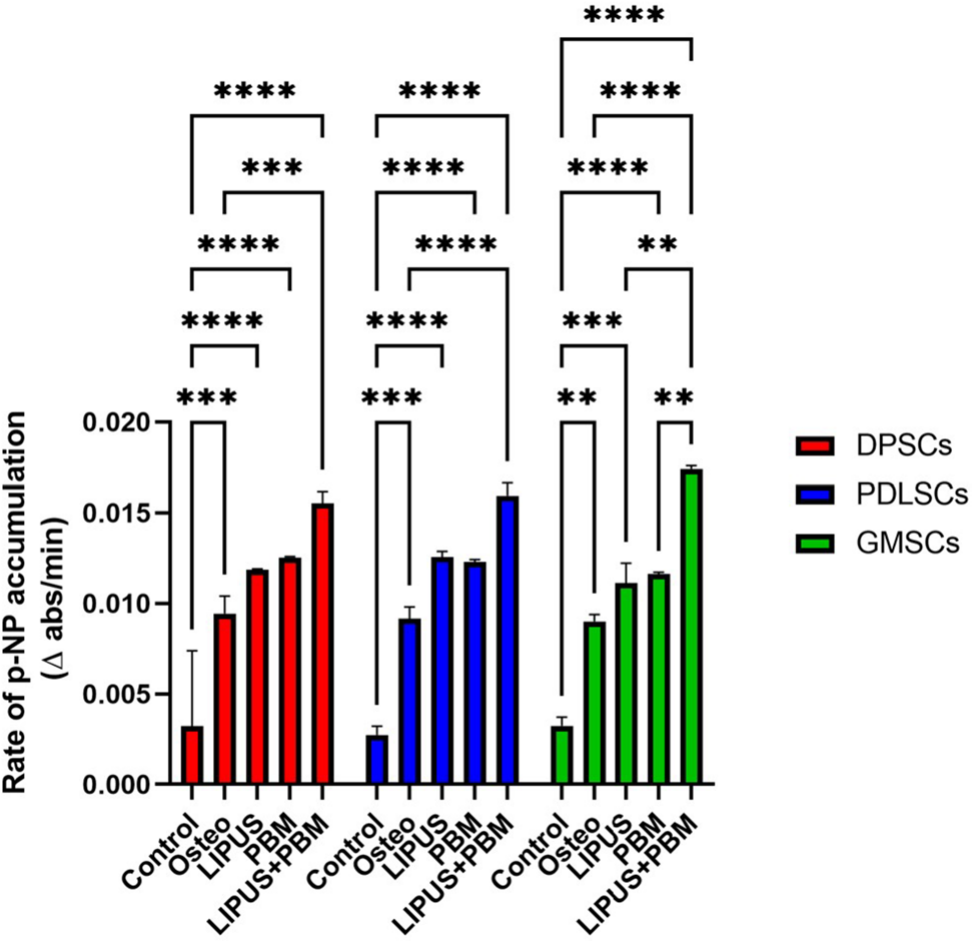
Mean and standard deviation of the rate of p-NP accumulation indicating ALP activity. *p < 0.05, **p < 0.01, ***p < 0.001, ****p < 0.0001 indicate significance for each group compared to the control and osteogenic groups and differences between the same stimulus method of groups

The ALP was significantly higher (*p* < 0.05) for the combined method than for the control and osteogenic groups in all three cell types. However, a nonsignificant difference (*p* > 0.05) was found between the laser and LIPUS methods in all cell types. The kinetic profile of the ALP assay demonstrating the accumulation of the yellow p-NP product over time among the different groups is shown in Fig. 6.

### PCR results

The results showed that when all three cell types were exposed to the combination of LIPUS and PBM, we observed a significant increase in the expression of all genes (*p* < 0.05) when compared to LIPUS and PBM individually.

However, it is noteworthy that *OPG* and *RUNX2* exhibited notably higher expression levels in the GMSCs group following the combined LIPUS and laser treatments. However, this difference was insignificant (*p* > 0.05) compared to the DPSCs and PDLSCs groups. On the other hand, regarding OC expression following the combined treatment, PDLSCs exhibited the highest expression level, and this difference was statistically significant (*p* < 0.05) compared to both the DPSCs and GMSCs groups. In contrast, the expression of *OC* in both PDLSCs and GMSCs showed no significant difference (*p* > 0.05). The expression of the odontogenic marker DSPP was significantly higher (*p* < 0.05) after combined stimulation of DPSCs compared to that of PDLSCs and GMSCs. On the other hand, the expression of DMP1 was not significantly different (*p* > 0.05) between the combined stimulation methods in different cells. The mRNA mean fold expression of all genes is shown in Table 4. Additional data are given in Online Resource 1.

**Table 4:** mRNA mean fold expression of *OPG, OC, RUNX2, DSPP,* and *DMP1*

	DPSCs	PDLSCs	GMSCs
***OPG***			
Control	1.05^A^ ± 0.04	1.10^A^ ± 0.01	1.11^A^ ± 0.05
Osteo	3.11^B^ ± 0.44	3.44^B^ ± 0.34	3.43^A^ ± 0.19
LIPUS	3.32^Ba^ ± 0.61	4.12^Bab^ ± 0.50	4.87^Bb^ ± 0.62
PBM	5.12^C^ ± 0.31	5.40^C^ ± 0.10	5.59^BC^ ± 0.52
LIPUS + PBM	6.39^D^ ± 0.78	5.98^C^ ± 0.45	6.74^C^ ± 0.86
***OC***			
Control	1.06^A^ ± 0.01	1.14^A^ ± 0.07	1.09^A^ ± 0.01
Osteo	2.63^B^ ± 0.15	2.26^A^ ± 0.19	2.43^A^ ± 0.69
LIPUS	6.37^Ca^ ± 0.87	3.21^Bb^ ± 0.18	3.75^Bb^ ± 0.64
PBM	7.16^CDa^ ± 0.63	4.28^Cb^ ± 0.09	4.13^Bb^ ± 0.09
LIPUS + PBM	8.25^Da^ ± 0.17	6.21^b^ ± 0.20	6.14^Cb^ ± 0.42
***RUNX2***			
Control	1.05^A^ ± 0.02	1.13^A^ ± 0.06	1.12^A^ ± 0.03
Osteo	2.91^B^ ± 0.44	3.11^B^ ± 0.42	2.61^AB^ ± 0.56
LIPUS	5.17^Ca^ ± 0.87	4.30^Bb^ ± 0.34	3.28^Bb^ ± 0.79
PBM	6.40^CDa^ ± 0.78	5.89^CDb^ ± 0.33	3.73^Cc^ ± 0.35
LIPUS + PBM	7.15^D^ ± 0.93	6.16^D^ ± 0.81	5.63^C^ ± 0.44
***DSPP***			
Control	1.01^A^ ± 0.01	1.02^A^ ± 0.01	1.02^A^ ± 0.01
Osteo	1.55^A^ ± 0.12	1.42^A^ ± 0.17	1.65^A^ ± 0.13
LIPUS	5.31^B^ ± 0.32	4.44^B^ ± 0.21	4.86^B^ ± 0.10
PBM	6.06^C^ ± 0.45	5.21^C^ ± 0.17	5.31^B^ ± 0.17
LIPUS + PBM	7.81^D^ ± 0.39	6.46^D^ ± 0.31	6.73^C^ ± 0.17
***DMP1***			
Control	1.03^A^ ± 0.02	1.02^A^ ± 0.02	1.02^A^ ± 0.01
Osteo	1.83^B^ ± 0.05	1.76^B^ ± 0.09	1.78^B^ ± 0.05
LIPUS	4.47^C^ ± 0.23	4.54^C^ ± 0.32	4.47^C^ ± 0.09
PBM	5.03^D^ ± 0.08	5.45^D^ ± 0.40	5.02^D^ ± 0.18
LIPUS + PBM	7.49^E^ ± 0.18	7.72^E^ ± 0.17	7.55^E^ ± 0.19

Different superscript capital letters in the same column indicate significant differences (*p* < 0.05); different superscript small letters in the same row indicate significant differences (*p* < 0.05)

## Discussion

PBM and LIPUS are well known for bio-stimulating MSCs in regenerative medicine. However, the synergistic effects of their dual application on dental MSCs (DMSCs) have not been extensively studied. There is no specific or standard protocol for the parameters used for the PBM application. PBM’s efficacy depends on many parameters, such as wavelength and intensity. Previous research has demonstrated that low light irradiation parameters, in the 600–1200 nm range, had a more favorable impact on tissue healing than high irradiation levels with varying degrees of penetration and biological effects [31, 36, 37]. In the present study, we tested the effect of a diode laser of 980 nm frequency, applied in a single dose for 60 s, on stem cell viability, migration, and osteo/odontogenic differentiation potential. This wavelength has been proven to have a biostimulatory effect on different MSCs sources [38, 39]. The current study showed increased cell viability of all irradiated stem cell types after 6 days. In addition, PBM improved the cell migration of all three cell types. These findings validate the viability results and agree with previous in vitro studies showing PBM to enhance MSC viability and migration [37, 40–44].

Numerous theories account for how PBM stimulates stem cell migration and proliferation. According to one theory, the mitochondrial energy from the laser is absorbed, driving the respiratory chain to generate more ATP and ultimately initiate mitosis by increasing RNA and protein synthesis [45]. In alignment, activating the mitochondrial respiratory chain boosts cell viability by releasing calcium into the cytoplasm, encouraging cell mitosis [46]. Another theory suggests that PBM results in the formation of reactive oxygen species (ROS), activating endogenous growth factors that drive stem cell proliferation and differentiation [47].

It is worth mentioning that laser-irradiated DPSCs and GMSCs showed slightly higher cell viability and migration levels than PDLSCs. This agrees with Santamaria et al. [48], who reported that GMSCs displayed a higher proliferation potential than PDLSCs under suboptimal proliferation conditions. This result suggests that GMSCs respond better to unfavorable culture conditions. Such flexibility of GMSCs, besides easy sourcing, represents an advantage for their use in therapeutic applications.

Similar to PBM, no specific LIPUS parameters are identified for bio-stimulation of stem cells. The optimal settings for these parameters can vary significantly depending on the type of cells, the desired outcome (e.g., cell proliferation vs. differentiation), and the specific tissue engineering application. In their study, Gao et al. (2015) [49] examined the disparities in the impact of LIPUS on the growth of mouse PDLSCs and DPSCs. It was found that an intensity of 750 mW/cm^2^ significantly enhanced the proliferation of DPSCs, whereas an intensity of 250 mW/cm^2^ strongly stimulated the proliferation of PDLSCs. The current study used LIPUS at an intensity of 750 mW/cm^2^. The biostimulatory effects of LIPUS on MScs can be attributed to the physical impact of ultrasonic vibrations through promoting changes in the biological features of the cell membrane, such as permeability and metabolite exchange [50–52].

When compared, the biostimulatory effect of PBM was found to be greater than that of LIPUS. Both DPSCs and GMSCs exposed to laser radiation had considerably increased proliferation rates compared to LIPUS-stimulated cells. These findings highlight the fact that the mechanism of action of PBM has a more significant influence on the cells' ability to proliferate. This finding emphasizes the hypothesis that MSCs derived from diverse sources exhibit biological characteristics that are niche- or site-specific. This could contribute to an explanation for why MSCs react differently to different stimuli [49, 53, 54].

On the other hand, combined stimulation methods using PBM followed by LIPUS resulted in higher significant cell viability values for all three cell types that were significant (*p* < 0.05) than PBM and LIPUS. This indicates that the combined stimulation was effective regardless of the cell type. In addition, the viability of GMSCs followed by PDLSCs was significantly higher (*p* < 0.05) than that of DPSCs. These findings endorse previous reports indicating the high proliferative capacity of GMSCs exceeding DPSCs and PDLSCs [48, 55, 56].

In the current study, MSCs were induced for osteogenic differentiation through culturing in osteogenic media for two weeks. Afterward, Alizarin red staining, ALP activity, and PCR to detect osteogenic and odontogenic markers were conducted to evaluate the differentiation. Our results showed that PBM has a stimulatory effect on promoting osteogenic differentiation of dental MSCs, as evident by calcium nodule formation, which was detected through Alizarin red staining. Furthermore, calcium nodule formation was significantly higher than that in the control and osteogenic media groups for all three cell types. These results agree with many studies on different types of stem cells, proving PBM’s ability to enhance osteogenic differentiation [40, 57]. Furthermore, Amid et al. [36] compared the exposure of DPSCs to PBM with two energies, 3 and 5 J/cm^2^. They concluded that PBM, especially at 3 J/cm^2^, as used in this study, enhanced the proliferation and osteogenic differentiation of DPSCs. On the other hand, DPSCs had a significantly higher nodule formation than PDLSCs and GMSCs, which showed similar results. This agrees with previous studies confirming the enhanced osteogenic potential of DPSCs compared to PDLSCs [58] and GMSCs [59].

The results of LIPUS stimulation revealed significantly higher levels of Alizarin Red S staining when compared to both the control and osteogenic groups. These findings align with previous research, which has consistently demonstrated the role of LIPUS in promoting osteogenic differentiation. Other studies have also reported similar outcomes, suggesting that LIPUS positively influences the differentiation of various types of stem cells toward osteogenesis [60, 61]. Furthermore, the mechanism underlying this osteogenic stimulation mediated by LIPUS has been linked to an increase in soluble RANKL [62].

A quantitative analysis assessed the enzymatic activity of alkaline phosphatase (ALP), a widely recognized early marker for cell differentiation leading to the synthesis of mineralized tissues, including dentin, enamel, and bone. Notably, ALP activity was significantly elevated in cells subjected to both PBM and LIPUS stimulation compared to the control and osteogenic control groups across all three types of cells studied. This observation aligns with findings from previous studies that also reported increased ALP activity in response to laser irradiation and LIPUS treatment [36, 60].

Interestingly, combining these two modalities resulted in a more substantial increase in ALP activity than PBM or LIPUS alone. This suggests that employing multiple forms of stimulation can synergistically accelerate the osteogenic differentiation process in these cells.

RT-qPCR results in the present study revealed that the osteogenic markers *OPG*, *OC*, *RUNX2*, and odontogenic markers *DSPP* and *DMP1* were upregulated in cells radiated with PBM and showed a significant difference (*p* < 0.05) compared with the control and osteogenic groups. The current RT‒PCR results align with several studies confirming that PBM enhances and upregulates the expression of osteogenic markers of dental stem cells [63, 64]. Cells responded differently to the markers; DPSCs showed significantly (*p* < 0.05) higher *OC* expression than PDLSCs and GMSCs. GMSCs showed higher *OPG* expression, while the expression of *DSPP* and *DMP1* did not show significant differences (*p* > 0.05) between irradiated cells. Many studies have revealed differences in osteogenic marker expression in different dental MSCs [58].

In response to LIPUS stimulation, there was an upregulation in the expression of osteogenic and odontogenic genes, although not to the same extent as observed with PBM. LIPUS was found to promote calcium deposition and enhance the production of osteogenic markers, including ALP, BMP2, OCN, OPG, and OPN [60, 65]. Hu et al. [61] conducted experiments with PDLSCs isolated from adult premolars exposed to varying levels of LIPUS. Their findings indicated that LIPUS significantly increased ALP activity, OCN expression, and the formation of calcified nodules. Moreover, it markedly elevated the expression levels of RUNX2 and integrin 1. Additionally, the RT-PCR results of this study demonstrated that DPSCs, PDLSCs, and GMSCs exhibited distinct expression profiles for pluripotent and differentiation markers, suggesting that MSCs derived from different tissues may possess tissue-specific biological properties. This could account for the varying responses of MSCs to different intensities of LIPUS [66].

Remarkably, when exposed to combined stimulation, DPSCs, PDLSCs, and GMSCs exhibited the highest expression levels of osteogenic and odontogenic markers. These gene expression outcomes underscored the synergistic effect of PBM and LIPUS in enhancing the osteogenic and odontogenic differentiation of dental MSCs. The results emphasize the uniqueness of gene expression in different cell types. Varying cell types have distinct responses to treatments, potentially influencing tissue engineering and regenerative medicine approaches that target specific cell populations. Furthermore, notable disparities in gene expression between the treatment and control groups emphasize the influence of treatments on regulating these genes.

In summary, the study indicates that the simultaneous use of PBM and LIPUS can enhance the results of different dental procedures requiring tissue regeneration and healing. Moreover, the uniform response of all three types of stem cells to the combined therapy underscores the adaptability of this method. Such versatility can streamline treatment methods and decrease the requirement for individualized stem cell selection, enhancing the accessibility and efficiency of cell-based regenerative dentistry. Furthermore, our study emphasizes the importance of carefully choosing the cell source in the field of regenerative dentistry. It is vital to note that selecting the appropriate cell source is crucial in tissue engineering procedures aimed at targeting tissue specificity.

Limitations of this study include the lack of a more extensive gene expression analysis to provide a deeper understanding of the molecular mechanisms underlying the observed benefit of combined PBM and LIPUS stimulation. Moreover, in vivo experiments are necessary to validate the findings of this study.

## Conclusions

The study demonstrates that the synergistic use of PBM and LIPUS significantly enhances the proliferation and differentiation properties of dental MSCs. These findings support their dual use in regenerative dentistry, particularly for dental and craniofacial tissue engineering. Moreover, the differential responses of DPSCs, PDLSCs, and GMSCs to PBM and LIPUS stimulation underscore the importance of cell source selection in cell-based tissue engineering strategies to target tissue specificity.

## Data Availability

The datasets generated during and/or analyzed during the current study are available from the corresponding author upon reasonable request.

## References

[1] Saber S. Tissue engineering in endodontics. J Oral Sci. 2009;5:495–507. 10.2334/josnusd.51.495. 10.2334/josnusd.51.495 20032600

[2] Paul K, Islam A, Volponi AA. Future horizons: embedding the evolving science of regenerative dentistry in a modern, sustainable dental curriculum. Br Dent J. 2022;232(4):207–10. 10.1038/s41415-022-3981-8. 35217737 10.1038/s41415-022-3981-8

[3] Battafarano G, Rossi M, De Martino V, Marampon F, Borro L, Secinaro A, et al. Strategies for bone regeneration: from graft to tissue engineering. Int J Mol Sci. 2021;22(3):1128. 33498786 10.3390/ijms22031128 PMC7865467

[4] Chen FM, Liu X. Advancing biomaterials of human origin for tissue engineering. Prog Polym Sci. 2016;53:86–168. 10.1016/j.progpolymsci.2015.02.004. 27022202 10.1016/j.progpolymsci.2015.02.004 PMC4808059

[5] Riha SM, Maarof M, Fauzi MB. Synergistic effect of biomaterial and stem cell for skin tissue engineering in cutaneous wound healing: a concise review. Polymers. 2021;13(10):1546. 34065898 10.3390/polym13101546 PMC8150744

[6] Song N, Scholtemeijer M, Shah K. Mesenchymal stem cell immunomodulation: mechanisms and therapeutic potential. Trends Pharmacol Sci. 2020;41(9):653–64. 10.1016/j.tips.2020.06.009. 32709406 10.1016/j.tips.2020.06.009 PMC7751844

[7] Wang M, Yuan Q, Xie L. Mesenchymal stem cell-based immunomodulation: properties and clinical application. Stem Cells Int. 2018;2018:3057624. 10.1155/2018/3057624. 30013600 10.1155/2018/3057624 PMC6022321

[8] Amato M, Santonocito S, Viglianisi G, Tatullo M, Isola G. Impact of oral mesenchymal stem cells applications as a promising therapeutic target in the therapy of periodontal disease. Int J Mol Sci. 2022;23(21):13419. 36362206 10.3390/ijms232113419 PMC9658889

[9] Kwack KH, Lee H-W. Clinical potential of dental pulp stem cells in pulp regeneration: current endodontic progress and future perspectives. Front Cell Dev Biol. 2022;10:857066. 10.3389/fcell.2022.857066. 35478967 10.3389/fcell.2022.857066 PMC9035692

[10] Fujii Y, Kawase-Koga Y, Hojo H, Yano F, Sato M, Chung UI, et al. Bone regeneration by human dental pulp stem cells using a helioxanthin derivative and cell-sheet technology. Stem Cell Res Ther. 2018;9(1):24. 10.1186/s13287-018-0783-7. 29391049 10.1186/s13287-018-0783-7 PMC5796442

[11] Mohan SP, Ramalingam M. Dental pulp stem cells in neuroregeneration. J Pharm Bioallied Sci. 2020;12(Suppl 1):S60–6. 10.4103/jpbs.JPBS_229_20. 33149432 10.4103/jpbs.JPBS_229_20 PMC7595495

[12] Zhou Z, Zheng J, Lin D, Xu R, Chen Y, Hu X. Exosomes derived from dental pulp stem cells accelerate cutaneous wound healing by enhancing angiogenesis via the Cdc42/p38 MAPK pathway. Int J Mol Med. 2022;50(6):5199. 10.3892/ijmm.2022.5199. 10.3892/ijmm.2022.5199 PMC9662140 36321793

[13] Andrukhov O, Behm C, Blufstein A, Rausch-Fan X. Immunomodulatory properties of dental tissue-derived mesenchymal stem cells: Implication in disease and tissue regeneration. World J Stem Cells. 2019;11(9):604–17. 10.4252/wjsc.v11.i9.604. 31616538 10.4252/wjsc.v11.i9.604 PMC6789188

[14] Yi Q, Liu O, Yan F, Lin X, Diao S, Wang L, et al. Analysis of senescence-related differentiation potentials and gene expression profiles in human dental pulp stem cells. Cells Tissues Organs. 2017;203(1):1–11. 10.1159/000448026. 27627434 10.1159/000448026

[15] Iezzi I, Cerqueni G, Licini C, Lucarini G, Mattioli BM. Dental pulp stem cells senescence and regenerative potential relationship. J Cell Physiol. 2019;234(5):7186–97. 10.1002/jcp.27472. 30362542 10.1002/jcp.27472

[16] de Freitas LF, Hamblin MR. Proposed mechanisms of photobiomodulation or low-level light therapy. IEEE J Sel Top Quantum Electron. 2016;22(3):348–64. 10.1109/jstqe.2016.2561201. 10.1109/JSTQE.2016.2561201 PMC5215870 28070154

[17] Heiskanen V, Hamblin MR. Photobiomodulation: lasers vs. light emitting diodes? Photochem Photobiol Sci. 2018;17(8):1003–17. 10.1039/c8pp90049c. 30044464 10.1039/c8pp00176f PMC6091542

[18] Yoo SH. Effect of photobiomodulation on the mesenchymal stem cells. Medical Lasers. 2020;9(2):119–25. 10.25289/ML.2020.9.2.119.

[19] Mohamad SA, Milward MR, Hadis MA, Kuehne SA, Cooper PR. Photobiomodulation of mineralisation in mesenchymal stem cells. Photochem Photobiol Sci. 2021;20(5):699–714. 10.1007/s43630-021-00047-5. 33945145 10.1007/s43630-021-00047-5

[20] Bölükbaşı Ateş G, Ak A, Garipcan B, Gülsoy M. Photobiomodulation effects on osteogenic differentiation of adipose-derived stem cells. Cytotechnology. 2020;72(2):247–58. 10.1007/s10616-020-00374-y. 32016710 10.1007/s10616-020-00374-y PMC7192995

[21] Kim HB, Baik KY, Seonwoo H, Jang K-J, Lee MC, Choung P-H, et al. Effects of pulsing of light on the dentinogenesis of dental pulp stem cells in vitro. Sci Rep. 2018;8(1):2057. 10.1038/s41598-018-19395-x. 29391502 10.1038/s41598-018-19395-x PMC5795010

[22] Latifa MA, Salah N, Sabry D, Abdelgwad M. Efficacy of photobiomodulation and vitamin D on odontogenic activity of human dental pulp stem cells. J Lasers Med Sci. 2021;12:30. 10.34172/jlms.2021.30. 10.34172/jlms.2021.30 PMC8558714 34733753

[23] Poolman RW, Agoritsas T, Siemieniuk RA, Harris IA, Schipper IB, Mollon B, et al. Low intensity pulsed ultrasound (LIPUS) for bone healing: a clinical practice guideline. BMJ. 2017;356: j576. 10.1136/bmj.j576. 28228381 10.1136/bmj.j576

[24] Uddin SMZ, Komatsu DE. Therapeutic potential low-intensity pulsed ultrasound for osteoarthritis: pre-clinical and clinical perspectives. Ultrasound Med Biol. 2020;46(4):909–20. 10.1016/j.ultrasmedbio.2019.12.007. 31959508 10.1016/j.ultrasmedbio.2019.12.007

[25] Lai WC, Iglesias BC, Mark BJ, Wang D. Low-intensity pulsed ultrasound augments tendon, ligament, and bone-soft tissue healing in preclinical animal models: a systematic review. Arthroscopy. 2021;37(7):2318-33.e3. 10.1016/j.arthro.2021.02.019. 33621647 10.1016/j.arthro.2021.02.019

[26] Jo J, Forrest ML, Yang X. Ultrasound-assisted laser thrombolysis with endovascular laser and high-intensity focused ultrasound. Med Phys. 2021;48(2):579–86. 10.1002/mp.14636. 33280145 10.1002/mp.14636 PMC9382677

[27] Zharov VP, Menyaev YA, Kabisov RK, Al’kov SV, Nesterov AV, Savrasov GV. Study on the design and application of combining low-frequency ultrasound with laser radiation in surgery and therapy. Crit Rev Biomed Eng. 2017;45(1–6):153–70. 10.1615/CritRevBiomedEng.v45.i1-6.80. 29953377 10.1615/CritRevBiomedEng.v45.i1-6.80

[28] Bakr M, Shamel M, Raafat S, Love R, Al-Ankily M. Effect of pulp capping materials on odontogenic differentiation of human dental pulp stem cells: an in vitro study. Clinical Exp Dental Res. 2023;10:1–13. 10.1002/cre2.816. 10.1002/cre2.816 PMC10860438 38053499

[29] Saber S, Raafat S, Elashiry M, El-Banna A, Schäfer E. Effect of different sealers on the Cytocompatibility and Osteogenic potential of human periodontal ligament stem cells: an in vitro study. J Clin Med. 2023;12(6):2344. 10.3390/jcm12062344. 36983344 10.3390/jcm12062344 PMC10056919

[30] Dahake PT, Panpaliya NP, Kale YJ, Dadpe MV, Kendre SB, Bogar C. Response of stem cells from human exfoliated deciduous teeth (SHED) to three bioinductive materials—an in vitro experimental study. Saudi Dent J. 2020;32(1):43–51. 10.1016/j.sdentj.2019.05.005. 31920278 10.1016/j.sdentj.2019.05.005 PMC6950838

[31] Alhazmi YA, Aljabri MY, Raafat SN, Gomaa SM, Shamel M. Exploring the effects of low-level laser therapy on the Cytocompatibility and Osteo/Odontogenic potential of gingival-derived mesenchymal stem cells: preliminary report. Appl Sci. 2023;13(14):8490.

[32] Ghorayeb SR, Patel US, Walmsley AD, Scheven BA. Biophysical characterization of low-frequency ultrasound interaction with dental pulp stem cells. J Ther Ultrasound. 2013;1(1):12. 10.1186/2050-5736-1-12. 25516801 10.1186/2050-5736-1-12 PMC4265945

[33] Sayed M, Mahmoud EM, Saber SM, Raafat SN, Gomaa SM, Naga SM. Effect of the injectable alginate/ nano-hydroxyapatite and the silica/ nano-hydroxyapatite composites on the stem cells: a comparative study. J Non-Cryst Solids. 2023;610:122327. 10.1016/j.jnoncrysol.2023.122327.

[34] Saber S, Gomaa S, Elashiry M, El-Banna A, Schafer E. Comparative biological properties of resin-free and resin-based calcium silicate-based endodontic repair materials on human periodontal ligament stem cells. Clinal Oral Investigations. 2023;27:6757–68. 10.1007/s00784-023-05288-5. 10.1007/s00784-023-05288-5 PMC10630253 37796335

[35] Elashiry M, Raafat S, Tay F, Saber S. Effect of rapamycin on human periodontal ligament stem cells that have been exposed to sodium hypochlorite. Life Sci. 2023;329:121989. 10.1016/j.lfs.2023.121989. 37524163 10.1016/j.lfs.2023.121989

[36] Amid R, Kadkhodazadeh M, Gilvari Sarshari M, Parhizkar A, Mojahedi M. Effects of two protocols of low-level laser therapy on the proliferation and differentiation of human dental pulp stem cells on sandblasted titanium discs: an in vitro study. J Lasers Med Sci. 2022;13: e1. 10.34172/jlms.2022.01. 35642237 10.34172/jlms.2022.01 PMC9131293

[37] Abo El-Dahab MM, Gheith M, Soliman NL, Aly RM. Effect of diode laser potentiality on proliferation of dental pulp stem cells (in vitro study). Bull Nat Res Cent. 2020;44(1):170. 10.1186/s42269-020-00414-9.

[38] Hanna R, Agas D, Benedicenti S, Ferrando S, Laus F, Cuteri V, et al. A comparative study between the effectiveness of 980 nm photobiomodulation delivered by hand-piece with gaussian vs. flat-top profiles on osteoblasts maturation. Front Endocrinol (Lausanne). 2019;10:92. 10.3389/fendo.2019.00092. 30842754 10.3389/fendo.2019.00092 PMC6391326

[39] Lazăr L, Manu DR, Dako T, Mâru MA, Suciu M, Ormenian A, et al. Effects of laser application on alveolar bone mesenchymal stem cells and osteoblasts: an in vitro study. Diagnostics (Basel). 2022;12(10):2358. 10.3390/diagnostics12102358. 36292047 10.3390/diagnostics12102358 PMC9600660

[40] Gutiérrez D, Rouabhia M, Ortiz J, Gaviria D, Alfonso C, Muñoz A, et al. Low-level laser irradiation promotes proliferation and differentiation on apical papilla stem cells. J Lasers Med Sci. 2021;12:e75. 10.34172/jlms.2021.75. 35155160 10.34172/jlms.2021.75 PMC8837851

[41] Feng J, Li X, Zhu S, Xie Y, Du J, Ge H, et al. Photobiomodulation with 808-nm diode laser enhances gingival wound healing by promoting migration of human gingival mesenchymal stem cells via ROS/JNK/NF-κB/MMP-1 pathway. Lasers Med Sci. 2020;35(8):1831–9. 10.1007/s10103-020-03040-z. 32451640 10.1007/s10103-020-03040-z

[42] Yin K, Zhu R, Wang S, Zhao RC. Low-level laser effect on proliferation, migration, and antiapoptosis of mesenchymal stem cells. Stem Cells Dev. 2017;26(10):762–75. 10.1089/scd.2016.0332. 28178868 10.1089/scd.2016.0332

[43] Ahrabi B, Rezaei Tavirani M, Khoramgah MS, Noroozian M, Darabi S, Khoshsirat S, et al. The effect of photobiomodulation therapy on the differentiation, proliferation, and migration of the mesenchymal stem cell: a review. J Lasers Med Sci. 2019;10(Suppl 1):S96-s103. 10.15171/jlms.2019.S17. 32021681 10.15171/jlms.2019.S17 PMC6983866

[44] Lee SH, Kim Y-J, Kim YH, Kim HY, Bhang SH. Enhancing therapeutic efficacy of human adipose-derived stem cells by modulating photoreceptor expression for advanced wound healing. Stem Cell Res Ther. 2022;13(1):215. 10.1186/s13287-022-02892-2. 35619187 10.1186/s13287-022-02892-2 PMC9137210

[45] Morsoleto M, Sella V, Machado P, Bomfim FD, Fernandes MH, Morgado F, et al. Effect of low power laser in biomodulation of cultured osteoblastic cells of Wistar rats1. Acta Cir Bras. 2019;34(2):e201900210. 10.1590/s0102-8650201900210. 30843943 10.1590/s0102-8650201900210 PMC6585914

[46] Sedlackova L, Korolchuk VI. Mitochondrial quality control as a key determinant of cell survival. Biochim Biophys Acta Mol Cell Res. 2019;1866(4):575–87. 10.1016/j.bbamcr.2018.12.012. 30594496 10.1016/j.bbamcr.2018.12.012

[47] Arany PR, Cho A, Hunt TD, Sidhu G, Shin K, Hahm E, et al. Photoactivation of endogenous latent transforming growth factor-β1 directs dental stem cell differentiation for regeneration. Sci Transl Med. 2014;6(238):238ra69. 10.1126/scitranslmed.3008234. 24871130 10.1126/scitranslmed.3008234 PMC4113395

[48] Santamaría S, Sanchez N, Sanz M, Garcia-Sanz JA. Comparison of periodontal ligament and gingiva-derived mesenchymal stem cells for regenerative therapies. Clin Oral Investig. 2017;21(4):1095–102. 10.1007/s00784-016-1867-3. 27270903 10.1007/s00784-016-1867-3

[49] Gao Q, Walmsley AD, Cooper PR, Scheven BA. Ultrasound stimulation of different dental stem cell populations: role of mitogen-activated protein kinase signaling. J Endod. 2016;42(3):425–31. 10.1016/j.joen.2015.12.019. 26830427 10.1016/j.joen.2015.12.019

[50] Tan Y, Guo Y, Reed-Maldonado AB, Li Z, Lin G, Xia SJ, et al. Low-intensity pulsed ultrasound stimulates proliferation of stem/progenitor cells: what we need to know to translate basic science research into clinical applications. Asian J Androl. 2021;23(6):602–10. 10.4103/aja.aja_25_21. 33818526 10.4103/aja.aja_25_21 PMC8577250

[51] Liu B, Chen W, Jiang J, Zhou W, Zhang Y, He R, et al. Treatment effect of low-intensity pulsed ultrasound on benzene- and cyclophosphamide-induced aplastic anemia in rabbits. Phys Ther. 2019;99(11):1443–52. 10.1093/ptj/pzz074. 31087076 10.1093/ptj/pzz074

[52] Mohaqiq M, Movahedin M, Mokhtari Dizaji M, Mazaheri Z. Upregulation of Integrin-α6 and Integrin-β1 gene expressions in mouse spermatogonial stem cells after continues and pulsed low intensity ultrasound stimulation. Cell J. 2018;19(4):634–9. 10.22074/cellj.2018.4286. 29105399 10.22074/cellj.2018.4286 PMC5672103

[53] Augello A, Kurth TB, De Bari C. Mesenchymal stem cells: a perspective from in vitro cultures to in vivo migration and niches. Eur Cell Mater. 2010;20:121–33. 10.22203/ecm.v020a11. 21249629 10.22203/ecm.v020a11

[54] Rasi Ghaemi S, Harding FJ, Delalat B, Gronthos S, Voelcker NH. Exploring the mesenchymal stem cell niche using high throughput screening. Biomaterials. 2013;34(31):7601–15. 10.1016/j.biomaterials.2013.06.022. 23871539 10.1016/j.biomaterials.2013.06.022

[55] Otabe K, Muneta T, Kawashima N, Suda H, Tsuji K, Sekiya I. Comparison of Gingiva, dental pulp, and periodontal ligament cells from the standpoint of mesenchymal stem cell properties. Cell Med. 2012;4(1):13–21. 10.3727/215517912x653319. 26858852 10.3727/215517912X653319 PMC4733865

[56] Subba TA, Varma S, Thomas B, Rao S, Kumar M, Talwar A, et al. Comparison of cellular and differentiation characteristics of mesenchymal stem cells derived from human Gingiva and periodontal ligament. J Int Soc Prev Community Dent. 2022;12(2):235–44. 10.4103/jispcd.JISPCD_259_21. 35462740 10.4103/jispcd.JISPCD_259_21 PMC9022390

[57] Sivakumar TT, Muruppel AM, Joseph AP, Reshmi A, Ramachandran R, Nair PD, et al. Photobiomodulatory effect delivered by low-level laser on dental pulp stem cell differentiation for osteogenic lineage. Lasers Dental Sci. 2019;3(3):175–81. 10.1007/s41547-019-00066-7.

[58] Kotova AV, Lobov AA, Dombrovskaya JA, Sannikova VY, Ryumina NA, Klausen P, et al. Comparative analysis of dental pulp and periodontal stem cells: differences in morphology, functionality, osteogenic differentiation and proteome. Biomedicines. 2021;9(11):1606. 10.3390/biomedicines9111606. 34829835 10.3390/biomedicines9111606 PMC8616025

[59] Lee KE, Kang C-M, Jeon M, Kim S-O, Lee J-H, Choi H-J. General gene expression patterns and stemness of the gingiva and dental pulp. J Dental Sci. 2022;17(1):284–92. 10.1016/j.jds.2021.02.012. 10.1016/j.jds.2021.02.012 PMC8739237 35028049

[60] Lim K, Kim J, Seonwoo H, Park SH, Choung PH, Chung JH. In vitro effects of low-intensity pulsed ultrasound stimulation on the osteogenic differentiation of human alveolar bone-derived mesenchymal stem cells for tooth tissue engineering. Biomed Res Int. 2013;2013:269724. 10.1155/2013/269724. 24195067 10.1155/2013/269724 PMC3806253

[61] Hu B, Zhang Y, Zhou J, Li J, Deng F, Wang Z, et al. Low-intensity pulsed ultrasound stimulation facilitates osteogenic differentiation of human periodontal ligament cells. PLoS ONE. 2014;9(4):e95168. 10.1371/journal.pone.0095168. 24743551 10.1371/journal.pone.0095168 PMC3990585

[62] Chiu CY, Tsai TL, Vanderby R Jr, Bradica G, Lou SL, Li WJ. Osteoblastogenesis of mesenchymal stem cells in 3-D culture enhanced by low-intensity pulsed ultrasound through soluble receptor activator of nuclear factor Kappa B Ligand. Ultrasound Med Biol. 2015;41(7):1842–52. 10.1016/j.ultrasmedbio.2015.03.017. 25922132 10.1016/j.ultrasmedbio.2015.03.017

[63] Abdelgawad LM, Abdelaziz AM, Sabry D, Abdelgwad M. Influence of photobiomodulation and vitamin D on osteoblastic differentiation of human periodontal ligament stem cells and bone-like tissue formation through enzymatic activity and gene expression. Biomol Concepts. 2020;11(1):172–81. 10.1515/bmc-2020-0016. 34233429 10.1515/bmc-2020-0016

[64] Rocha EAD, Alvarez MMP, Pelosine AM, Carrilho MRO, Tersariol ILS, Nascimento FD. Laser Photobiomodulation 808 nm: effects on gene expression in inflammatory and osteogenic biomarkers in human dental pulp stem cells. Front Pharmacol. 2022;12:782095. 10.3389/fphar.2021.782095. 35111053 10.3389/fphar.2021.782095 PMC8802107

[65] Costa V, Carina V, Fontana S, De Luca A, Monteleone F, Pagani S, et al. Osteogenic commitment and differentiation of human mesenchymal stem cells by low-intensity pulsed ultrasound stimulation. J Cell Physiol. 2018;233(2):1558–73. 10.1002/jcp.26058. 28621452 10.1002/jcp.26058

[66] Augello A, De Bari C. The regulation of differentiation in mesenchymal stem cells. Hum Gene Ther. 2010;21(10):1226–38. 10.1089/hum.2010.173. 20804388 10.1089/hum.2010.173

